# A dual regimen of ritonavir/darunavir plus dolutegravir for rescue or simplification of rescue therapy: 48 weeks’ observational data

**DOI:** 10.1186/s12879-017-2755-4

**Published:** 2017-09-30

**Authors:** Amedeo F. Capetti, Maria Vittoria Cossu, Giancarlo Orofino, Gaetana Sterrantino, Giovanni Cenderello, Giuseppe V. De Socio, Anna Maria Cattelan, Alessandro Soria, Stefano Rusconi, Niccolò Riccardi, Gian Maria Baldin, Fosca P. Niero, Giorgio Barbarini, Giuliano Rizzardini

**Affiliations:** 1 1st Division of Infectious Diseases, ASST Fatebenefratelli-Sacco, Via Giovanni Battista Grassi, 74, pavillion 56, Malattie Infettive, 2nd floor, 20157 Milan, Italy; 2 1st Division of Infectious Diseases Amedeo di Savoia Hospital, Torino, Italy; 3 Division of Infectious Diseases, “Careggi” Hospital, Firenze, Italy; 4 Division of Infectious Diseases, Ospedali Galliera, Genova, Italy; 5 Infectious Diseases Clinic, Azienda Ospedaliero-Universitaria di Perugia, Perugia, Italy; 6 Infectious and Tropical Diseases, Azienda Ospedaliera-Universitaria di Padova, Padova, Italy; 7 Clinic of Infectious Diseases, San Gerardo Hospital, ASST Monza, University of Milano-Bicocca, Monza, Italy; 8 Infectious Diseases Clinic, DIBIC Luigi Sacco, University of Milano, Milano, Italy; 9 Infectious Diseases Clinic, “San Martino” Hospital, Genova, Italy; 10 2nd Division of Infectious Diseases, Università Cattolica del Sacro Cuore, Rome, Italy; 11 2nd Division of Infectious Diseases, “Policlinico San Matteo” Hospital, Pavia, Italy; 12 Whitwaterstrand University, Johannesburg, South Africa

**Keywords:** Dolutegravir, Darunavir, Ritonavir, Switch, Dual, Salvage, Simplification

## Abstract

**Background:**

Dolutegravir (DTG) plus darunavir/ritonavir (DRV/r) is a simple combination of drugs that has the best genetic barrier to HIV-1 resistance and may be fit for salvage therapy.

**Methods:**

All HIV-1-infected subjects treated with DTG plus DRV/r between March 2014 and September 2015 in eight Italian centres were included in the analysis. The main metabolic data, efficacy parameters and safety data routinely collected were provided. This observational study is aimed to assess the efficacy of such approach. The primary end-point was the proportion of subjects achieving or maintaining virologic suppression <50 copies/mL at week 24. Secondary end points were maintaining virologic suppression in the follow-up (weeks 48 and 96) and safety.

**Results:**

One hundred and thirty subjects were followed for a median of 56 months. Reasons for switching were simplification (44.6%), viral failure (30%), toxicity (16.9%), non-adherence (4.6%), persistent low-level viremia (3.1%), and drug-drug interaction (0.8%). At baseline, 118 subjects had documented resistance to 1 to 5 antiretroviral classes while 12 had viral rebound at a time when genotypic tests were not yet available.

Seventeen and 14 subjects took DRV/r and DTG twice daily, respectively. One subject was lost to follow-up, one discontinued for liver enzymes’ elevation, one died of illicit drug abuse and one of cancer-related complications.

The proportion of subjects with ongoing HIV replication dropped from 40% to 6.1%. Those with undetectable viral load increased from 38.5% to 76.2%. At week 48, 17.7% had HIV RNA between 1 and 49 copies/mL.

The number of subjects with altered serum glucose, creatinine, ALT, AST, total-, HDL- and LDL-cholesterol, triglycerides and MDRD <90 mL/min decreased by week 48, while those having MDRD <60 mL/min remained 4.6%. Overall 90/283 baseline laboratory alterations returned to normality.

**Conclusions:**

Switching to DTG plus DRV/r proved to be safe, suppressing viral replication without metabolic impact.

**Electronic supplementary material:**

The online version of this article (10.1186/s12879-017-2755-4) contains supplementary material, which is available to authorized users.

## Background

The present work is the 48-week update of a previously reported study [1], concerning a retrospective-prospective follow-up of subjects who had been switched for any reason to a dual combination of dolutegravir (DTG) plus darunavir/ritonavir (DRV/r). A randomized clinical trial comparing the switch to this regimen towards the continuation of DRV/r plus 2 nucleoside analogues is still recruiting patients [2]. When we started this strategy of dual rescue therapy or simplification of more complex rescue regimens, the OPTIONS study, the first randomized trial to utilize a web utility in combination with centralized expert opinion to guide the selection of salvage antiretroviral therapy was published [3]. The 413 participants had antiretroviral (ARV) experience or resistance to nucleoside reverse transcriptase inhibitors (NRTI) and non-NRTI (NNRTI), were taking a protease inhibitor (PI)-containing regimen for at least 8 weeks prior to study entry, and had plasma HIV RNA ≥1000 copies/mL. Enfuvirtide (ENF), etravirine (ETR), DRV, maraviroc (MVC), raltegravir (RAL), and tipranavir (TPV) plus ritonavir booster allowed 20 potential ARV regimens. Fifty-three subjects had obliged regimen choice due to poor phenotypic sensitivity score while 360 were randomized to add or not NRTIs. The resulting regimens were rather complex, with 3 to 6 drugs and twice-daily dosing, and generally contained DRV and RAL. The proportion of patients achieving viral suppression at 48 weeks (<50 HIV-1 RNA copies/mL) was 70.2% and 74.1% for the Omit NRTI and for the Add NRTI arms, respectively [4]. Prescribers were generally oriented to prefer simpler regimens.

The combination of elvitegravir/cobicistat/tenofovir alafenamide/emtricitabine plus 800 mg DRV (two pills once daily) demonstrated superiority towards the continuation of baseline therapy in a randomized (2:1), open-label, switch trial in treatment-experienced virologically suppressed subjects [5]. The baseline regimens contained in median 5 pills per day and the primary endpoint was maintaining <50 HIV-1 RNA copies/mL.

In this context some authors were looking forward for nucleoside-sparing regimens in the long-term care of HIV infection, welcoming the possible positive contribution of DTG [6].

The present study reports what the clinical practice had already realised, aiming for convenience, simplicity, potency and high genetic barrier, prescribing this combination to several patients and controlling them closely until viral suppression was achieved.

## Methods

All subjects who had started DTG plus DRV/r between March 1, 2014 and September 30, 2015 were included in an Italian observational multicenter cohort named Tivista (*Tiv*icay plus Prez*ista* Observational cohort). After approval by Ethics Committees (EC) no further enrolment was allowed. Since the last publication two centres that were waiting for EC approval joined the group, increasing the population size. For homogeneity no data were collected after week 48 (plus 8 weeks’ window as observational studies do not dictate strict timelines). The follow-up was censored at November, 30, 2016. Subjects were not required to be naive to DRV or DTG, but regimens containing both drugs were not included.

Participating subjects have signed an informed consent according to local procedures, the study has been approved by the coordinating centre and by all participating centres and conducted according to the Good Clinical Practice (GCP) Guidelines.

The aim of the study is to assess the efficacy of this approach. The primary end-point was the proportion of subjects achieving or maintaining virologic suppression <50 copies/mL at week 24. Secondary end points were maintaining virologic suppression in the follow-up (weeks 48 and 96) and safety, as proportion of drop-outs for any reason and grade 3–4 adverse events.

### Safety data and laboratory standards

In this update it was decided to document the impact of the switch on some routine safety analyses. Investigators retrospectively reported to the coordinating center serum glucose, alanine aminotransferase (ALT), aspartate aminotransferase (AST), total cholesterol (TC), high density lipoprotein (HDL-C), low density lipoprotein (LDL-C) and triglycerides (TG). The estimated glomerular filtration rate (e-GFR) was calculated at baseline and at follow-up according to the Modification of Diet in Renal Disease (MDRD) equation [7]. Plasma viral suppression status was classified as harbouring ≥50 HIV-1 RNA copies/mL, or detectable below 50 copies/mL, or undetectable (no virus detected, NVD = 0 copies/mL).

Viremia was measured depending on centers with Abbot HIV-1 RT-PCR, threshold 37 copies, COBAS TaqMan HIV-1 test, threshold 20 copies, and Single Copy Assay. TruGene™ was used for resistance testing, validated on the Stanford Algorithm, and cumulative data was considered. Data were collected from electronic or hand-written patients’ case record forms, according to each centre’s organization and sent as pre-specified excel files. Available data from routine therapeutic monitoring of darunavir and dolutegravir trough plasma concentration were analysed.

For the toxicity analyses the Common Terminology Criteria for Adverse Events (CTCAE, Version 4.03, June 14, 2010) was considered [8]. Adverse clinical events and deaths were reported to the local Ethics Committees and authorities as required by the law.

### Statistical analysis

The statistical analysis was limited to routine parameters (median, mean and standard error) and to the paired t-test for the comparison between the baseline and 48-week metabolic and immunologic values. The virologic response is reported as the number of patients in each viral suppression category and as the median and interquartile range of log_10_ copies/mL in those who do not reach suppression at each time point. The other analyses consider the on-treatment population. The sample size was obtained from the participants’ adhesion.

## Results

**Fig. 1:**
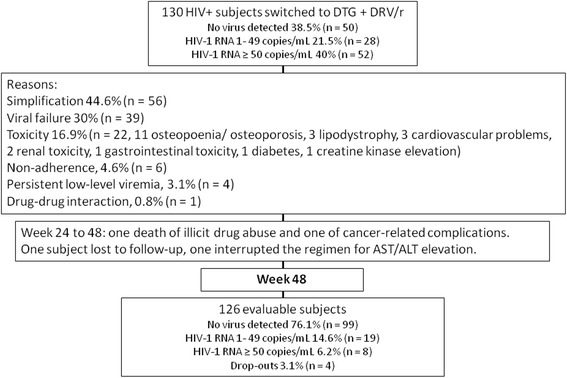
Algorithm of the study population. DTG = dolutegravir; DRV/r = darunavir boosted with ritonavir; AST = aspartate aminotransferase; ALT = alanine aminotransferase

One hundred and thirty subjects were followed for a median of 56 months, mean 64 months. The median age was 52 years, females were 25.4% and non-Caucasians 8.9%. The median baseline CD4+ T-cell count was 526/mmc (IQR 329–718). Fifty-nine subjects had been exposed to INSTIs and all had been exposed to PIs. Eighty-one had darunavir in their pre-switch regimen and one had dolutegravir. The main risk factor for acquiring HIV was being male homosexual, 48.5% (*n* = 63), followed by drug abuse, 26.9% (*n* = 35) and heterosexual intercourse, 24.6% (*n* = 32). Reasons that led to the switch were simplification, 44.6% (*n* = 56), viral failure 30% (*n* = 39) toxicity 16.9% (*n* = 22, of which 11 for osteopoenia/osteoporosis, 3 each for lipodystrophy and cardiovascular problems, 2 for renal toxicity and one each for gastrointestinal toxicity, diabetes and creatine kinase elevation), non-adherence, 4.6% (n = 6), persistent low-level viremia, 3.1% (*n* = 4), and drug-drug interaction, 0.8% (*n* = 1). The algorithm of the study population is represented in Fig. 1.

At baseline 118 subjects had documented resistance to 1 to 5 ARV classes. For the remaining 12 patients, viral failure was not accompanied by a genotypic test. Twenty-three subjects (17.7%) had never experienced virologic failure but had transmitted drug resistance mutations.

One hundred and sixteen patients (89.2%) had NRTI resistance-associated mutations (RAMs) at baseline, 98 (75.4%) had NNRTI RAMs, 91 (70%) had PI RAMs and 12 (10.6%) had integrase strand transfer inhibitors (INSTI) RAMs (See Table 1).

**Table 1:**
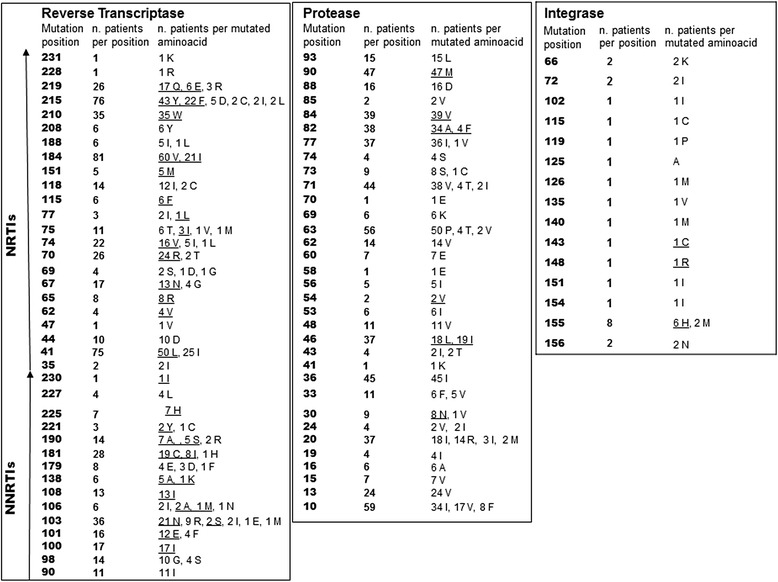
Baseline HIV-1 drug resistance mutations in the study population

Primary resistance mutations (Wensing, 2017) are underlined

Twenty subjects had reduced baseline sensitivity to darunavir (Stanford median score 15, range 15–40), and 12 had reduced sensitivity to INSTIs (Stanford median score 10, range 10–25) but none rebounded. None developed new drug resistance mutations during the study.

Of those subjects who had resistance to 3–4 drug classes (*n* = 70), 35 were not failing at baseline, but rather simplified from raltegravir and boosted darunavir-based three- (*n* = 31) or four-drugs regimens (*n* = 9).

One hundred and thirteen have started the combination of DRV 800 mg plus RTV 100 mg plus DTG 50 mg every 24 h, 3 DRV 600 mg plus RTV 100 mg every 12 h and 14 DRV 600 mg plus RTV 100 mg plus DTG 50 mg every 12 h. All of those who were on BID therapy had active HIV-1 replication at baseline (range 5.46–6.16 log_10_ HIV-1 RNA copies/mL). All of them suppressed viremia to <50 copies/mL during the follow-up.

Between week 24 and 48 one subject was lost to follow-up, one interrupted the regimen for liver enzymes’ elevation, one died of illicit drug abuse and one of cancer-related complications.

**Fig. 2:**
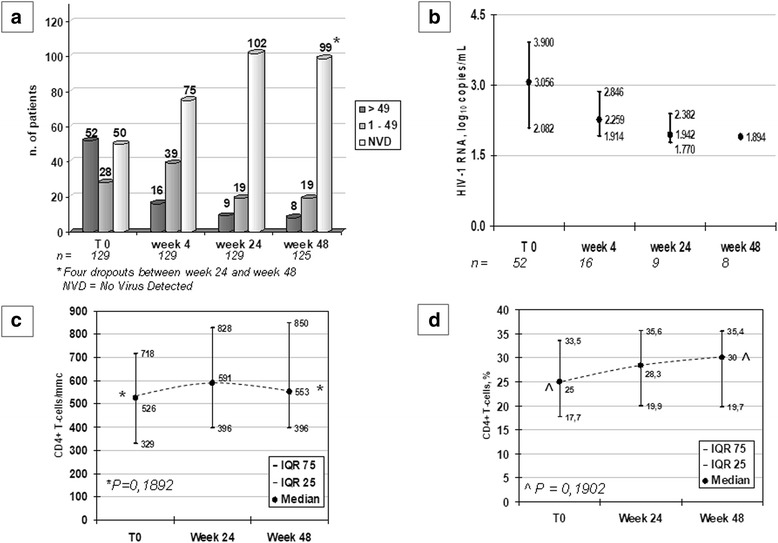
Virologic and immunologic response. **a** n. of patients by HIV-1 RNA, copies/mL; **b** HIV-1 replication decay in the population with ongoing viral replication, log_10_ copes/mL; **c** CD4+ T-lymphocytes/mmc; **d** CD4+ T-lymphocytes, %. eGFR = estimated Glomerular Filtration Rate, calculated on the Modified Diet for Renal Disease (MDRD) score; AST = aspartate aminotrasferase; ALT = alanine aminotrasferase; TC = total cholesterol; HDL-C = HDL cholesterol; LDL-C = LDL cholesterol

The proportion of subjects harboring active HIV replication dropped from 40% at baseline to 6.1% by week 48, while those who had NVD at real-time polymerase chain reaction (RT-PCR) increased from 38.5% to 76.2%. Overall, at baseline 78 subjects had <50 HIV-1 RNA copies/mL (60%) and this proportion increased at week 48 to 90,8% (*n* = 118) At week 48, 19 subjects (14,6%) had HIV RNA between 1 and 49 copies/mL and eight subjects (6,1%) had ≥50 HIV-1 RNA copies/mL. Of the latter, 3 had a slow but constant decay from median baseline 5.12 to 2.30 log_10_ HIV-1 RNA copies/mL, 3 had at least three-class drug resistance with a Stanford DRV score > 60 and median viral decay from 4.39 to 1.75 log_10_ HIV-1 RNA copies/mL, and 2 had dropped out of therapy 4 and 7 days before blood sampling. Considering separately two populations, those who started with active HIV replication (≥ 50 copies/mL) and those who started with 0–49 copies/mL, at week 48 the success rate was 88.5% in those who had started with active replication, as 3 with high baseline viral load and 3 with extensive baseline resistance, had still very low but detectable viremia, while in those who started with 0–49 copies/mL the success rate was 97.4% as in this group two subjects were not on therapy on the sampling day, as described above. Of interest, both were in the subgroup starting with 1–49 copies/mL, as some authors suggest that this low but persistent viremia may sometime hide adherence problems. The mean viral load in this population at week 48 was 1.89 log_10_ copies/mL (range 1.70–2.57, Fig. 2). The CD4+ T-cell absolute count and proportion increased by 27 cells/mm^3^, +5%, not reaching statistical significance.

A single-point pharmacokinetic analysis in a subgroup of 32 subjects confirmed the safety of the association: DTG median C_trough_ was 579 ng/mL (range 275–5036), while DRV median C_trough_ was 3007 ng/mL (range 678–8053) and no values were found to be below the safety threshold. Five subjects were taking DRV 600 mg twice daily and had C_min_ above the threshold for resistant strains, and three were taking DTG twice daily.

**Fig. 3:**
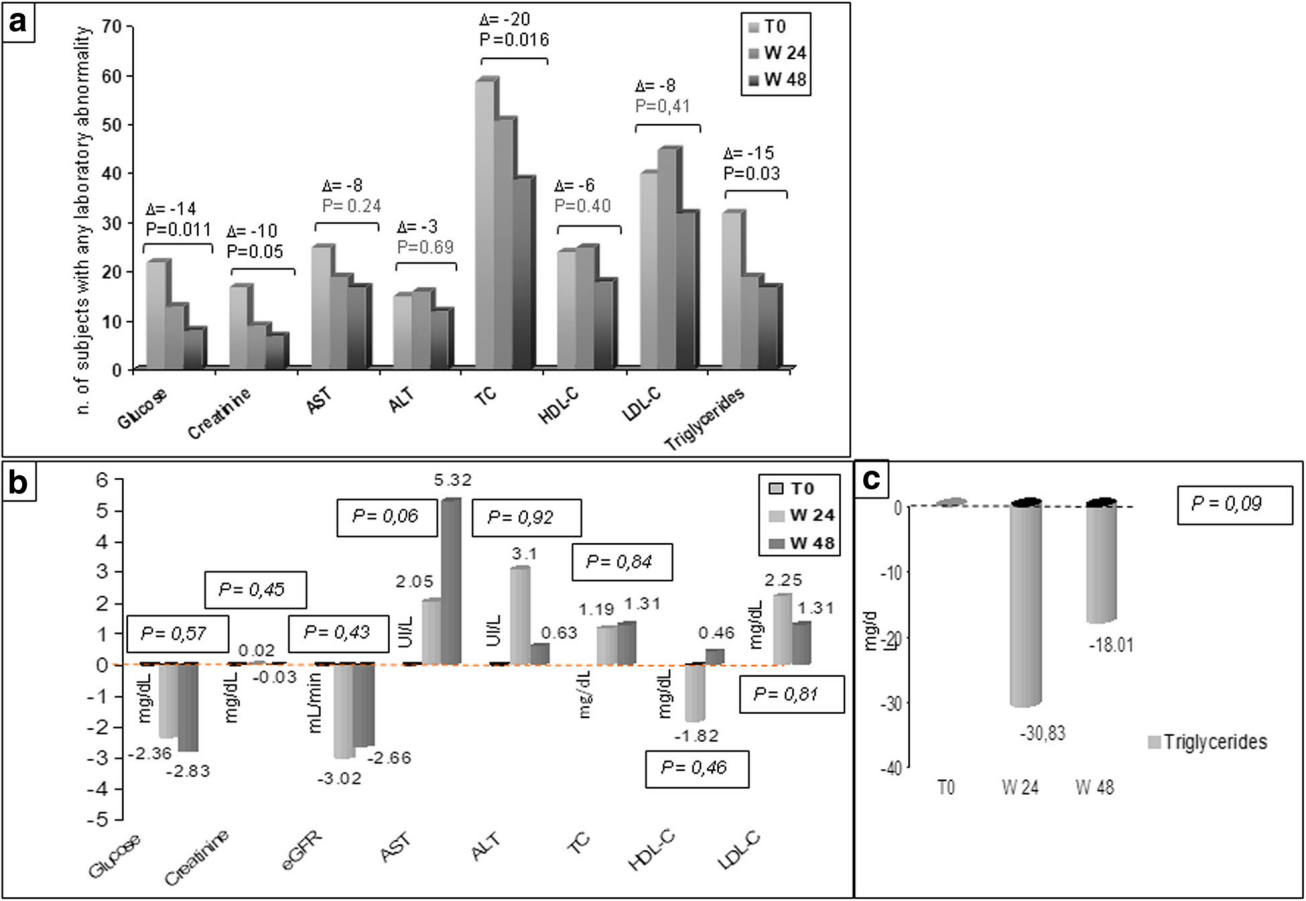
Metabolic profile during the follow-up. **a** Number of subjects with any laboratory abnormality at baseline, week 24 and week 48. **b** Variation of metabolic indicators from baseline, mean values; **c** Variation in triglycerides (different scale). AST = Aspartate Aminotransferase; ALT = Alanine Aminotransferase; HDL-C = High-Density Lipoprotein - Cholesterol; LDL-C = Low-Density Lipoprotein - Cholesterol; MDRD = Modified Diet for Renal Disease; TC = Total Cholesterol

The proportion of subjects having any metabolic alterations at baseline decreased for serum glucose, creatinine, MDRD <90 mL/min, ALT, AST, total-, HDL- and LDL-cholesterol and triglycerides. The proportion of subjects having MDRD <60 remained stable at 4.6%. Overall 84/175 (48%) baseline laboratory alterations returned to normality. Considering each parameter in the whole population, none varied significantly (Fig. 3).

In particular, the median variation in serum creatinine was −0,03 and the eGFR decrease on average by 2.66 mL/min. None of the subjects who had altered serum creatinine at baseline showed progression and 6 out of 8 improved, having discontinued tenofovir.

Overall this strategy yielded mean savings for € 169,428.00 considering the whole population, the observation period and the dropouts. The figure was generated only from the cost of the previous and current antiretroviral regimen.

## Discussion

While awaiting the results of the randomized DUALIS study [2], our data suggest that the combination of DTG plus DRV/r may be considered reliable in salvage or simplification of salvage regimens. The pharmacokinetic interaction between the two drugs [9] did not seem to impact neither causing subtherapeutic C_min_ in the subjects tested nor leaving space for plasma HIV-1 RNA rebound in any subject. In particular, 70% of our patients would not have been suitable for the combination of DTG plus atazanavir, which has a favourable interaction profile [10] due to baseline resistance to the latter and it should be said that clinical experience with this combination is still limited though promising. Moreover, we have not reported significant neurological side effects, as suggested by Hoffman et al. [11] Possible reasons may be the combination, which does not include abacavir, the fact that many of our patients had received the indication to take the drug in the morning and the relatively small number of other at risk subjects (15 elderly and 33 women).

The study limitations include poor patient selection (the only two inclusion criteria were being HIV-1 antibody positive and having started DTG plus DRV/r by the end of September 2015), leading to the inclusion of a heterogeneous population, made out of failures and simplification, once- and twice-daily regimens, adherent and poorly adherent subjects and subjects harbouring treatment-resistant and treatment-sensitive strains. The set of metabolic parameters furthermore reflects standard follow-up data shared by centres and does not include bone density measures nor inflammatory markers.

## Conclusions

Switching to DTG plus DRV/r provided a simple and safe rescue regimen to all subjects, controlling viral replication in a high proportion of patients. The metabolic impact was favourable in a proportion of subjects who had baseline alterations, while no significant modifications were observed in mean and median values when also normal baseline values were included.

## Additional file

Additional file 1: Tivista Study Week 48 database. (TIFF 31 kb)

## Abbreviations

ALT: Alanine aminotransferase
ARV: Antiretroviral
AST: Aspartate aminotransferase
CD4: Cluster difference 4
CTCAE: Common Terminology Criteria for Adverse Events
C_trough_: Trough concentration
DRV/r: Darunavir boosted with ritonavir
DTG: Dolutegravir
EC: Ethics Committee
eGFR: Estimated glomerular filtration rate
ENF: Enfivirtide
ETV: Etravirine
GCP: Good clinical practice
HDL: High-density lipoprotein
HIV-1: Human immunodeficiency virus type 1
LDL: Low density lipoprotein
MDRD: Modified diet for renal disease
MVC: Maraviroc
NNRTI: Non-nucleoside reverse transcriptase inhibitor
NRTI: Nucleoside reverse transcriptase inhibitor
NVD: No virus detected
PI: Protease inhibitor
RAL: Raltegravir
RNA: Ribonucleic acid
RT-PCR: Real-time Polymerase Chain Reaction
TPV: Tipranavir
